# Modeling the Impact of Intermittent Preventative Treatment on the Spread of Drug-Resistant Malaria

**DOI:** 10.1371/journal.pmed.0030181

**Published:** 2006-04-04

Until the mid-20th century, malaria occurred in most temperate, subtropical, and tropical countries of the world. Then, the introduction of powerful insecticides, including DDT, made it possible to eliminate this human parasitic disease in many temperate countries by controlling the mosquitoes that transmit malarial parasites between people. Elsewhere, eradication efforts were less successful, but the use of inexpensive antimalarial drugs such as chloroquine and sulfadoxine-pyrimethamine (SP) further reduced global morbidity and mortality from malaria. Sadly, the rapid spread of resistance to chloroquine (and more recently to SP) has resulted in a resurgence of malaria over the past three decades. Nowadays, 40% of the world's population is at risk of contracting malaria, and every year, it kills at least 1 million people—mainly children. Pregnant women and their unborn children are particularly vulnerable to malaria, for whom it is a major cause of perinatal mortality, low birth weight, and maternal anemia.

One way to reduce malaria morbidity and mortality is to treat asymptomatic individuals, regardless of their infection status, with regular therapeutic doses of antimalarial drugs. Intermittent preventative (or presumptive) treatment (IPT) is currently used in pregnant women (IPTp) in malaria-endemic areas, and IPT for infants (IPTi) is also being considered. However, before an intervention of this type is widely introduced, its potential impact on the spread of drug-resistant parasites needs to be investigated. A badly designed intervention could increase the speed at which malaria parasites become resistant to new drugs, an outcome that public health officials want to avoid. Ideally, such information would come from field trials, but in practice such trials are rarely undertaken, so researchers, including Wendy Prudhomme O'Meara, David Smith, and Ellis McKenzie, have turned instead to mathematical modeling. O'Meara and colleagues now describes a model that has allowed them to evaluate the possible impact of IPTp and IPTi on the spread of drug-resistant malaria parasites. Their analysis highlights the importance of carefully choosing which drugs to use for IPTi, and indicates which conditions are most likely to encourage the spread of drug resistance.

Drug use patterns—how quickly the body removes each drug, how well an individual's immune response deals with malaria parasites, and how often each person gets bitten by an infected mosquito (the transmission intensity)—all affect the spread of drug-resistant parasites. Prudhomme O'Meara and colleagues built these factors into a composite model that incorporates a human and a parasite population model. They then used their model to predict the potential for drug-resistant parasites to spread in low- and high-transmission settings, and to predict how the use of IPT in adults and infants, the time taken for drug elimination, and the treatment of infections (instead of asymptomatic individuals alone) might affect the spread of drug resistance.

One prediction of their model is that whereas fully resistant parasites (which can survive a full therapeutic dose of an antimalarial drug) are more likely to spread under conditions of high transmission, partially resistant parasites (which survive at intermediate drug concentrations) are more likely to spread in low-transmission areas, a result supported by epidemiological observations. The model also predicts that the use of a drug for IPT to which there is no existing resistance in a high-transmission area will accelerate the appearance of partial resistance, followed by an explosion of full resistance. Another analysis indicates that drugs that are rapidly eliminated from the body (e.g., chlorproguanil-dapsone) may be preferable to those that linger (e.g., SP). This latter type of drug maximizes the period of protection from each treatment but also maximizes the time when enough drug is present to allow selection of resistant parasites (the window for selection). Finally, comparing IPTp with IPTi, the model predicts that partially resistant parasites will spread faster when IPT is given to infants (who have little or no immunity to malaria) than when given to adults (who often are immune to some degree).

The researchers stress that their model provides a qualitative, not a quantitative, assessment of how partial and fully resistant malaria parasites will spread in different communities under different drug use strategies. But, they say, the model can be used as a tool to determine the critical questions that need to be addressed before broad implementation of IPT. In particular, they note, their model highlights the importance of carefully selecting the drug to be used in IPTi programs in different settings so that protection is maximized while minimizing the chances of antimalarial drug resistance emerging.

